# Translational biophotonics with Raman imaging: clinical applications and beyond

**DOI:** 10.1039/d1an00954k

**Published:** 2021-10-01

**Authors:** Isaac J. Pence, Conor L. Evans

**Affiliations:** Wellman Center for Photomedicine, Harvard Medical School, Massachusetts General Hospital 149 13th Street Charlestown Massachusetts 02129 USA Evans.Conor@mgh.harvard.edu

## Abstract

Clinical medicine continues to seek novel rapid non-invasive tools capable of providing greater insight into disease progression and management. Raman scattering based technologies constitute a set of tools under continuing development to address outstanding challenges spanning diagnostic medicine, surgical guidance, therapeutic monitoring, and histopathology. Here we review the mechanisms and clinical applications of Raman scattering, specifically focusing on high-speed imaging methods that can provide spatial context for translational biomedical applications.

## Abstract 2

The technological developments of high-speed spontaneous and coherent Raman scattering based imaging for translational applications including surgical guidance, histopathology, and pathophysiological monitoring.

## Graphical abstract

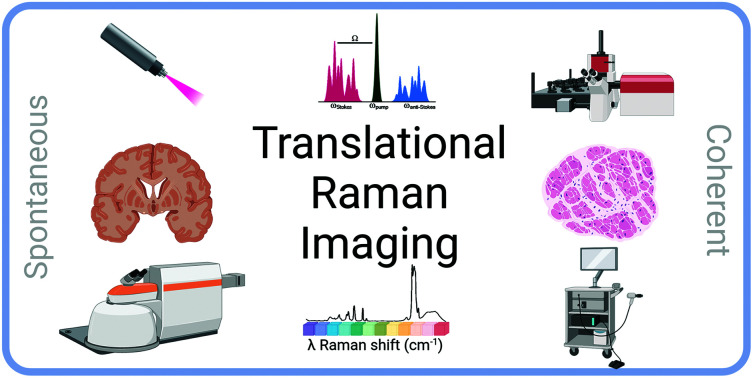

## 1 Introduction

In his 1930 Nobel lecture, Sir C. V. Raman alluded to the discoveries and developments in comprehending the interactions between light and matter as a starting point in the development of a new branch of knowledge.^1^ Inspired by the colors he saw in the waters of the Mediterranean Sea, years of exploration led to his discovery of the Raman effect. This fundamental curiosity, to explore the interactions of light with matter, has continued to drive the development of vibrational imaging technologies from the realms of physics and chemistry to new advances and applications in biology and medicine.

**Fig. 1:**
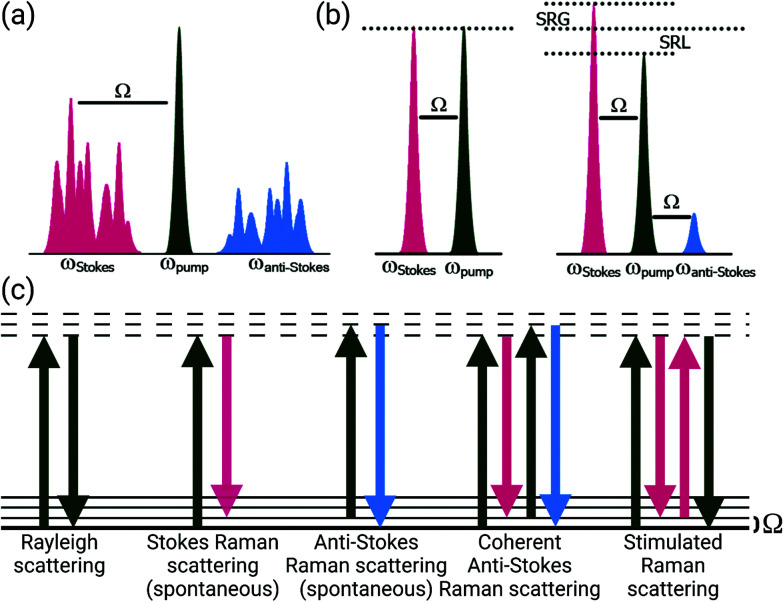
Spectral and energy level diagrams for spontaneous Raman scattering, coherent anti-Stokes Raman scattering (CARS), and stimulated Raman scattering (SRS). (a) Excitation of a sample with a single frequency causes spontaneous Stokes and anti-Stokes Raman scattering frequencies. (b) Coherent excitation using pump and Stokes frequencies tuned to a vibration (*Ω*) generates both CARS and SRS (gain or loss, depending upon instrumentation) signals when pulses are appropriately modulated and overlapped in space and time. (c) Energy level transitions for Rayleigh scattering and both spontaneous and coherent Raman scattering processes. Created with BioRender.com.

Raman scattering was first described in 1928 as “new type of secondary radiation” appearing as a “modified scattered radiation of degraded frequency” that accompanies the diffuse radiation scattered from molecules in dust-free liquids and gases.^2^ Using strongly converging sunlight to illuminate the samples, this report described the inelastic scattering of light in material. The Raman effect occurs with the creation of an induced dipole moment in a molecule, causing an instantaneous transition to a virtual state; this process enables coupling between vibrational states and the transfer of a quantum of vibrational energy with the molecule (Fig. 1). This transferred quantum of energy causes a shift in the wavelength of the scattered light relative to the incident beam (*Ω*) and is highly specific to the molecular bond and the physico-chemical properties of the sample, such that extremely precise information about the composition, state, and polarization of a material can be determined. Furthermore, the intensity of spontaneous Raman scattering is proportional to the concentration of the scattering species, yielding the potential for quantitative analysis. Years after the advent of the laser, stable light sources were used to demonstrate this compositionally-specific scattering in biological specimens.^3^

Early research groups using Raman scattering *in vivo*^4–6^ were able to demonstrate the molecular specificity of this technique for characterizing anatomical differences and discriminating diseases. These studies primarily utilized point-based measurements either with fiber optic probes or with confocal microscopes for data acquisition due to the long integration times needed to obtain adequate signal contrast. In general, spontaneous Raman scattering is a weak or infrequent process that has rendered image acquisition impractical for *in vivo* applications or visualizing dynamic processes. This lack of spatial information has been a challenge for Raman scattering methods and has required the development of numerous advances that combine new techniques and innovations in order to make Raman imaging a reality.

The intrinsic sensitivity of the Raman effect to both organic and inorganic moieties has been applied to a wide array of fields for analyses. The breadth of applications have been recently reviewed, spanning archaeology & art,^7^astronomy,^8^ food & agriculture,^9,10^ forensics,^11^ geology,^12^ industrial combustion,^13^ and pharmaceutical development,^14–16^ and have continually expanded as the instrumentation for spectroscopic studies has become more widely available.

A wide range of biological and medical investigations have applied Raman spectroscopic techniques to cells, animal models, biofluids, and tissues (both *ex vivo* and *in vivo*).^17–20^ While many promising analytical methods for biomarker development, pathophysiological assessment, and diagnostic applications have been described *ex vivo*, most investigations continue to utilize point-based spectroscopic measurements, which provide limited *spatial* context into the molecular variations present in these biological samples. Raman imaging approaches comprise a valuable advancement in the assessment of biomedical samples with non-destructive label-free contrast.^21,22^ Despite the promise of Raman imaging for numerous applications, the fundamentally weak spontaneous Raman scattering process leads to long acquisition times for microspectroscopic imaging applications. As such, there have been numerous investigations into technological and analytical advancements that can dramatically increase the generated Raman signal or efficiently acquire data at high-speeds to propel these techniques towards clinical utility. Ultimately, the factor of greatest importance is the speed by which actionable biochemical information can be obtained and conveyed for clinical decision making. As a non-destructive and non-invasive tool that does not require labels for molecularly specific detection and has the ability to provide this information within the context of an image with respect to cells and tissues, Raman scattering methods still hold great promise to address challenges in translational biomedicine, but high-speed imaging has been a major hurdle for the field. This targeted review highlights developments in Raman imaging for translational applications, targeting advances that have been implemented to increase detection and imaging speeds within a spatial context for intraoperative *ex vivo* or clinical *in vivo* imaging.

## 2 Spontaneous Raman scattering methods

Most translational demonstrations of Raman scattering are based on fiber optic probes to interface with tissue. While spectra can be rapidly (≤50 ms per measurement) obtained from samples, specimens, and patients, these point-based measurements provide little to no spatial context for the comparison of samples; this lack of spatial information has been a challenge for translation. Furthermore, interpretation of the spectroscopic data within a clinical setting is hampered by the complex spectral signatures obtained, which likely has contributed to the limited adoption of these methods despite the extensive field of research addressing clinical applications.^18,23^ The combination of distinct imaging approaches and advanced analysis algorithms developed, however, have demonstrated the potential to extend the feasibility of spontaneous Raman scattering methods for clinical and intraoperative translation.

### 2.1 Raman microspectroscpic imaging methods

**Fig. 2:**
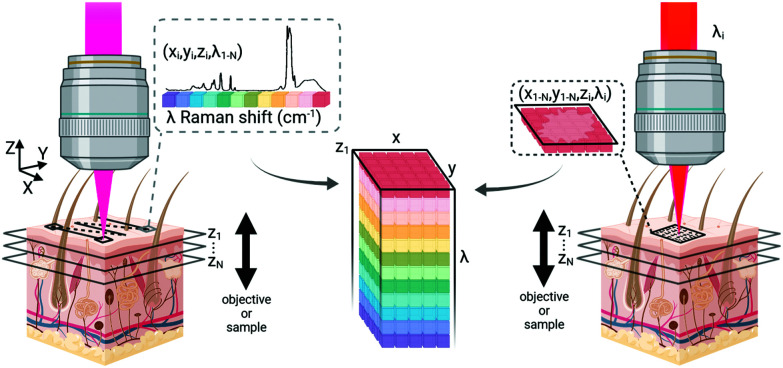
Data acquisition for Raman scattering microscopy. Confocal Raman microscopy (left) typically acquires a spectrum at each point on a sample plane, temporarily dwelling in position for data collection while scanning across the tissue to generate data. Coherent Raman imaging (right) rapidly scans excitation light across a sample plane to generate an image of a single Raman shift frequency. Spectral information is gathered by changing the beat frequency between the pump and Stokes beams before acquiring additional images. Both approaches can generate volumetric data by moving the axial focus of the beam with respect to the sample surface, however the axial resolution of confocal imaging is lower than that of nonlinear imaging methods. Created with BioRender.com.

As many biomedical imaging applications of Raman scattering are interested in cellular changes associated with disease, Raman microspectroscopic imaging has often been utilized for the analysis of cells and tissues. This approach is typically based on confocal laser scanning microscope designs where the beam is raster scanned across the specimen to acquire a full spectrum at each location (Fig. 2, left). In some cases, sample scanning methods may also be employed, where the laser focus remains stationary while the sample itself undergoes motion. Due to the low Raman scattering efficiencies of most biological samples, this approach is time consuming and can take numerous hours to acquire spectral images, depending on the inherent Raman scattering signal-to-noise ratio (SNR), the chosen spatial and spectral resolution, and the sample size. These time requirements typically render such approaches incompatible with clinical and intraoperative implementation. Long-duration measurements are often accomplished with continuous wave laser sources, depositing moderate amounts of energy into the sample to generate the desired Raman scattering. Raman scattering in most cells and tissues is also accompanied by background scattering (*e.g.* Rayleigh) and autofluorescence, the latter of which can be minimized by utilizing longer wavelength sources that are less likely to efficiently excite the electronic transitions in molecules that generate fluorescence. These longer excitation wavelengths, often near-infrared (NIR) at 785 nm or 830 nm, fall within an optical window in most biological samples where the absorption from most chromophores is reduced and facilitates greater photon penetration for deeper interrogation. This, however, can be a costly trade-off as the efficiency of the Raman scattering phenomenon is inversely proportional to wavelength (follows 1/*λ*^4^). In all cases, high resolution spontaneous Raman imaging requires the careful selection of excitation sources from the available, stable lasers needed for experiments.

**Fig. 3:**
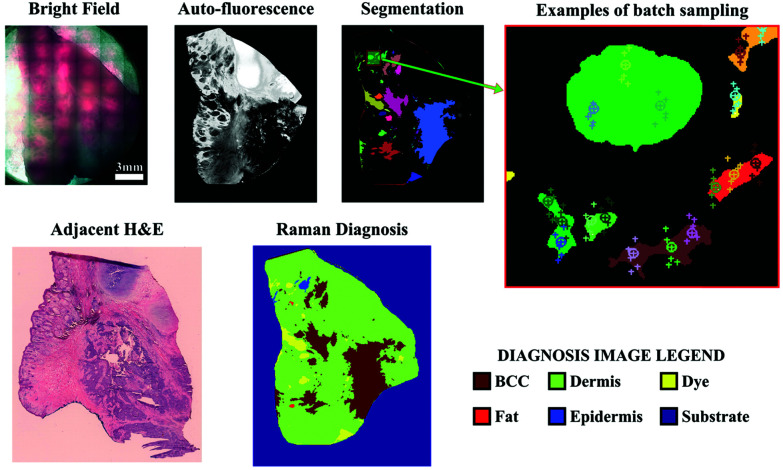
Automated multiplexed multimodal spectral imaging diagnosis of skin tissue using segment-averaged spectra. The expanded view of the segmented image shows an example of batch sampling points with markers of the same color belonging to the same batch. Each batch of six spectra was acquired in 2 s. Reproduced under the terms of Creative Commons Attribution 4.0 licence, Sinjab *et al.*, Tissue diagnosis using power-sharing multifocal Raman micro-spectroscopy and auto-fluorescence imaging, *Biomed. Opt. Express*, 2016, **7**(8), 2993–3006, DOI: 10.1364/BOE.7.002993.^28^

Even with optimized system hardware, measurement of a single spectrum from a single sample site can take hundreds of milliseconds or more. This time requirement greatly restricts spontaneous Raman imaging approaches to stable *ex vivo* samples such as tissues prepared for histological analysis. Efforts have been made to reduce imaging times to more clinically-relevant durations and include line-scanning, multifocal, or wide-field imaging approaches^24^ along with predictive-methods for sparse spatial sampling,^25^ the combination of on-line chemometrics,^26^ or integration with other imaging modalities such as autofluorescence imaging.^27^ One promising demonstration of this augmented Raman data collection method combined multimodal spectral imaging of tissue autofluorescence with multifocal Raman microspectroscopy.^28^ By using rapid autofluorescence imaging to acquire spatial maps and predict segmentation, spatial light modulation to multiplex Raman excitation, and digital micromirror devices to replace the conventional fixed spectrometer slit with a programmable binary array, automated segmentation and imaging of nearly 800 points of interest on a single excised skin sample (∼1 cm^2^) could be achieved in approximately 11 minutes (Fig. 3). The imaging speeds achieved through selective sampling methods for data acquisition have enabled the translation of these approaches for intraoperative analysis for skin and breast cancer surgical applications, which have been recently reviewed in detail by Lizio *et al.* as part of this themed collection on Biomedical Raman Imaging.^29^ The developments briefly described here comprise a major enhancement in the data acquisition and imaging speed of spontaneous Raman imaging systems. The continued developments of these microspectroscopic methods to efficiently characterize samples within a few minutes or less have significant potential to bring Raman techniques forward for clinical applications.

### 2.2 Fiber optic probe and SERS-based methods

Microscopic approaches can obtain subcellular resolution at the cost of long integration times and high fluence rates. However, there have been alternative intraoperative investigations based on non-destructive Raman scattering methods to balance imaging time and sampling performance. Several groups have accomplished this balance through the combination of widefield imaging and Raman fiber optic probe tracking to quickly acquire Raman spectral data with a spatial resolution that is suited for surgical guidance. For example, Yang *et al.* demonstrated the use of a computer-vision approach to track the position of a Raman probe and its laser spot on a sample to calculate measurement position. This approach was used to generate an overlay of spectral mapping that was projected onto a simultaneously captured brightfield image.^30^ In this demonstration, the authors pre-defined the specific tissue components of interest and used pre-recorded spectra for each to generate an color image (RGB) overlay of specific sample locations that correspond to a particular tissue type. In a similar approach, by adding simple fiducial markers onto a typical handheld Raman probe, Horgan *et al.* were able to demonstrate in *ex vivo* and *in vivo* animal studies that digital probe tracking could be used to define a tumor tissue margin through the combined use of Raman spectral measurements and exogenous fluorescence detection.^31^ For surgical guidance, the use of a handheld tool is familiar for surgeons assuming the technique is compatible with sterilization protocols and can be integrated into standard practice to provide new information. Extending this method for clinical implementation can have greater requirements on probe localization and imaging resolution, depending on the application of interest. Skin tumor demarcation, for example, typically uses a relatively wide excision margin to minimize the chance of recurrence, however this margin is determined based upon the location of the lesion. Alternatively, brain cancer excision requires a narrow margin to preserve critical structures and precise determination of measurement position is typically compared against pre-operative magnetic resonance imaging. In a study of 17 patients undergoing surgical excision of brain tumors, Jermyn *et al.* used intraoperative tracking of a Raman probe to acquire and overlay *in situ* spectral measurements from various locations that demonstrated this point-based detection could differentiate tissue type and glioma grade with high sensitivity and specificity.^32^ This work and that of other research groups suggests that these tools could be used for guidance and tissue differentiation in the operating theater to provide biochemical information during procedures.

Other approaches have investigated the use of low spatial resolution Raman fiber probe based images to study excised tissue after removal as a means to map tissue margins and guide surgical procedures. Determining whether a sufficient margin of healthy tissue has been used for surgical excision requires the ability to determine the tissue composition as a function of depth from the exposed specimen surface. Spatially offset Raman spectroscopy (SORS) based methods have been investigated for probing depth specific information by altering the separation between the light source and detector, with numerous applications in biomedicine.^33^ One such implementation for intraoperative imaging of breast tumor margins utilized a large area SORS probe design that acquired spectra from the surface and several hundered micrometers of superficial depths of excised tissue following lumpectomy.^34^ By mounting the *ex vivo* tissue on an automated tissue scanning system that rotated the sample and positioned the SORS probe, sparse spatial sampling of the superficial tissue could be mapped. Raman spectral signatures that differed by composition were determined using multivariate analysis and enabled the identification of spatial regions corresponding to a particular disease status. While this approach does not convey the high spatial resolution of other imaging approaches, this 3D scanner-based SORS imaging approach has the potential for intraoperative excised margin assessment within a clinically-relevant timescale. An alternative fiber probe design combining point-based Raman spectroscopic measurements with fluorescence lifetime imaging was also reported for *ex vivo* tissue mapping, here using both Raman mapping and fluorescence signals to guide spectral acquisition with the potential for *in vivo* application.^35^ The use of guided spatial sampling introduces a balance in utilizing spontaneous Raman scattering approaches for imaging within the constraints of clinically relevant time-frames, a limitation conferred due to the relative weakness of the spontaneous Raman scattering phenomenon.

To circumvent the need for lengthy integration times that prohibit clinical Raman imaging studies, approaches that increase the scattered signal intensity have also been investigated. Surface-enhanced Raman scattering (SERS) is an optical phenomenon exhibited by the interaction of Raman-active molecules with specifically-crafted surfaces of noble metals that form “hot spots”. Confined to this highly localized volume of interaction, the surface plasmon resonance (and chemical charge-transfer) can lead to Raman scattering intensity enhancements ≥10^14^ fold greater than those observed from spontaneous Raman scattering. SERS enhancement can be gained from nanoscale surfaces, found on either patterned structures^36^ or nanoparticles,^37^ with nanoparticles being a preferred means of delivering probes within cells and tissues.^38^ A wide range of bespoke SERS structures have been created to boost the Raman scattering intensity, including spheres,^39,40^ rods,^41^ bowties,^42,43^ stars^44,45^ and other shapes.^46^ Nanoparticles for SERS can be readily targeted using antibodies, peptides, and aptamers to reach specific cellular and tissue compartments.^38^

**Fig. 4:**
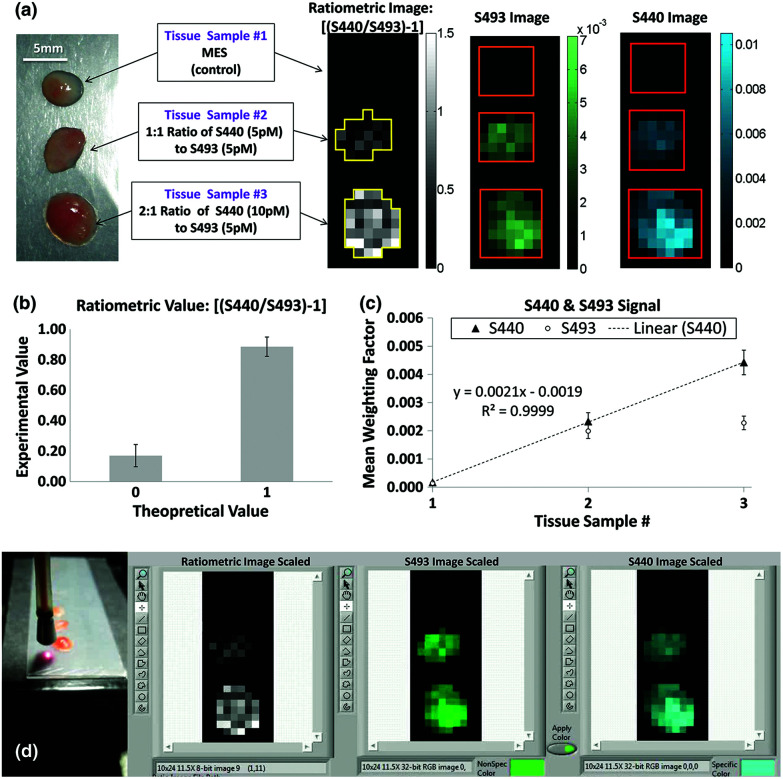
(a) The experimental design (left) using human colon tissue biopsies incubated in MES, a 1 : 1 ratio of 5 pM of S440 and S493, or a 2 : 1 ratio of 10 pM of S440 and 5 pM of S493. The resulting ratiometric values of the tissue samples after raster scan are shown on the right. Outlines of the ROIs used for analysis are shown in yellow. (b) Quantification of the ratiometric values within the ROIs as shown in (a), right. The error bars are standard errors of the mean, *σ*/√*n*, where *n* is the number of pixels within each of the ROIs: *n* = 21 for the ratiometric value of 0 and *n* = 35 for the ratiometric value of 1. (c) Quantification of the S440 and S493 signal within the ROI as shown in (a), right. The error bars are the standard error of the mean. (d) Image of the probe placed for imaging with respect to the sample and capture of software interface for experimental data acquisition. Reprinted from Garai *et al.*, High-sensitivity, real-time, ratiometric imaging of surface-enhanced Raman scattering nanoparticles with a clinically translatable Raman endoscope device, *J. Biomed. Opt.*, 2013, **18**(9), DOI: 10.1117/1.JBO.18.9.096008, with permission under a Creative Commons Attribution 3.0 License.^48^

Many SERS investigations rely on point-wise spectral measurements to localize the enhanced signals within tissue sections after topical application. The need for excitation and collection of Raman scattered light, as well as the delivery of SERS structures, limits most clinically-oriented studies to superficial applications such as endoscope compatible evaluation.^47^ Furthermore, the combinatorial approach of utilizing a widefield imaging modality such as MRI, PET, or fluorescence in combination with point-wise SERS measurements has been further investigated. With regards to directly imaging SERS signals, Garai *et al.* developed a SERS imaging fiber bundle assembly intended to acquire ratiometric images from tissue with both targeted and untargeted nanoparticles (Fig. 4).^48^ Designed to acquire images at a distance within 15 mm of the tissue surface, this device has been demonstrated with *ex vivo* human colon cancer samples, generating low spatial resolution images encoding a mixture of SERS targets. While intended for clinical compatibility, this system, along with many other SERS designs, faces hurdles related to biodistribution, cytotoxicity, and regulatory approval for *in vivo* human applications.

SERS enhancement can also be applied to SORS for surface-enhanced spatially offset Raman scattering, or SESORS.^49^ The substantial signal enhancement offered by SERS nanoparticles is highly advantageous for SORS detection, and can enable improved imaging contrast. Recent efforts applying SESORS to animal cancer models have been successful in achieving imaging using benchtop setups.^50^ Current efforts focused on building endoscopic SESORS systems may offer new methods for intraoperative Raman imaging. However, as it makes use of nanoprobes similar to those of SERS, SESORS methods are likely to face similar regulatory barriers for future clinical applications.

An alternative implementation for SERS imaging is applied to intraoperative histology. By topically applying the signal-enhancing nanoparticles only after a specimen has been excised, SERS has been applied in or adjacent to the operating theater to provide rapid evaluation of cell surface biomarkers using molecular specific SERS particles for breast cancer lumpectomy procedures.^51^ Utilizing a combination of targeted and untargeted nanoparticles applied to the surface of an excised tumor, the margin of the tissue is mounted on a stage and a custom-designed imaging probe is scanned across the tissue, mapping the mixture of SERS spectral signatures which can subsequently be demultiplexed to generate tissue maps of various biomarker targets and generate a binary image of healthy and tumor tissue at the specimen surface. Given the signal enhancement and multiplex ability afforded by using SERS particles that can be specifically functionalized to bind known cancer cell surface markers, this approach provides the means to look at the molecular composition of *ex vivo* samples in a time-frame consistent with intraoperative workflow for surgical guidance without the limitations associated with introduction of SERS particles to humans *in vivo*.

Fiber optic probe based methods for detecting Raman scattering, either alone or in combination with other rapid imaging approaches, have the potential to provide biochemical information to characterize a tissue *in vivo* or a specimen *ex vivo*. However, these approaches generally trade spatial resolution to obtain imaging speeds that are required for clinical applications. Furthermore, many of these point-based methods rely on contact measurements that can deform the tissue while conferring stability to the measurement location. Non-contact probe measurements are relatively insensitive to small movements as the spatial information is not highly resolved, however stability of an imaging system and movement of a sample are paramount considerations during microscopic imaging. Especially for *in situ* imaging on live samples, mechanical isolation and stabilization are critical. Low frequency movements caused by respiration or pulsatile blood flow can significantly alter the focus on a microscopic level. Often, increasing imaging speed to a rate that is faster than these low frequency movements can help mitigate their influence. Accounting for movement between imaging frames may be possible after data acquisition based on registration of image fiducial markers. This approach, however, can require dense spatial mapping that often requires increased imaging time. Imaging instruments also often require several components with fans for cooling, which generate higher frequency vibrations that may also factor into movements that must be carefully controlled. Dynamic modification of the light focused onto a sample can be performed with the appropriate choice of technology (adaptive optics, laser ranging, *etc.*) however each of these components must be carefully considered. Biomedical specimens are often of interest because of dynamic changes which further require imaging systems to acquire data at a high enough rate to capture changes on a relevant time scale. This combination of fast, stable imaging at a high enough spatial resolution to visualize and quantify changes is a challenge for translational imaging systems, often resulting in large devices that dampen vibrations and provide mechanical stability for optical paths, but which may be incompatible with the clinical environment. The methods and advancements described above for increasing imaging speed of Raman scattering methods at microscopic spatial resolutions indicate the potential for numerous clinical applications, however, for some dynamic processes, further advances are still necessary.

## 3 Coherent Raman imaging

Spontaneous Raman methods provide considerable molecular information, however, the inherent weakness of the Raman signal stands as a major barrier to many imaging applications. While surface-enhanced Raman methods boost the Raman signal strength, the requirement for exogenous nanoscale structures poses regulatory issues for clinical adoption and translation. An ideal approach would comprise enhanced Raman signal strength for *in vivo* applications without the need for exogenous materials.

Coherent Raman methods provide video-rate, non-destructive, chemically-specific imaging of tissues without the need for exogenous labels for contrast. Coherent Raman imaging (CRI) has been developed as a viable tool set that continues to grow as a field for translation from the laboratory bench to clinical and intraoperative settings.^52–55^ Based on the nonlinear mixing of two (or more) ultrafast laser sources, Raman-active vibrations can be selectively driven in a coherent manner by tuning the difference in the frequency (*ω*_*Δ*_) of the excitation sources, pump (*ω*_pump_) and Stokes (*ω*_Stokes_), to match the vibrational resonance (*Ω*) of the target molecule (Fig. 1). To achieve the needed nonlinear mixing, pico- or femtosecond pulsed lasers with short duty cycles are used for nonlinear excitation with low average power. The combined beams can be rapidly scanned over the tissue to generate images (Fig. 2, right). The multiphoton nature of CRI gives rise to a rapid fall off in photon density as the distance from the focal plane increases, providing inherent 3D sectioning and depth selectivity in thick samples without excess heat generation or photochemical sample damage.^56,57^ While many nonlinear imaging tools have been investigated, coherent anti-Stokes Raman scattering (CARS) and stimulated Raman scattering (SRS) imaging methods are the most commonly used given both their practicality and inherent sensitivity to Raman features that enable species separation and identification. To achieve the nonlinear interactions of the copropagating laser sources, high photon densities are facilitated using high numerical aperture (NA) microscope objectives to strongly focus the light. While not intrinsically a challenge for investigations of cells *in vitro* or thin sections of *ex vivo* tissue, coherent Raman scattering can be strongly directional, and differs between modalities: transmission mode measurements can be advantageous for both CARS and SRS carried out in thin samples, however, for many biological applications, epi-collection is a necessity. By relying upon the multiple linear scattering events in turbid samples, epi-collection for these techniques has been demonstrated with differing levels of success.^52,58^ Furthermore, these CRI methods offer the potential for quantitative imaging of sample constituents; while CARS displays a quadratic relationship between signal intensity and concentration, SRS signals are linear, allowing direct visualization of composition and structure for quantitative image analysis.

### 3.1 Intraoperative coherent Raman histopathology

CRI methods offer approximately 10 000 times greater signal strength over spontaneous Raman for micron-sized objects, converting acquisition times of hours with spontaneous Raman imaging to only fractions of a second. This dramatic speed improvement has been realized for tissue mapping, and several instruments and applications have been described for tissue analysis within or adjacent to the operative theater. A major benefit to CRI based optical imaging is the removal of the need for time consuming staining and sample processing, the limited volume of biopsy tissue that is eventually examined, and the restriction of specific tissue features to those for which staining protocols are well-established. A recent targeted review of SRS histopathology and computationally-aided diagnostics covers some of the these topics in detail for further insight on the topic as part of this themed collection on Biomedical Raman Imaging.^59^

Initially demonstrated by Freudiger *et al.*, *ex vivo* tissues that have been freshly excised can be imaged at multiple vibrational bands to provide contrast akin to stain-free histopathology using CRI. In an initial report, SRS was implemented instead of CARS to remove the contribution of non-resonant background signals that can degrade image contrast, and imaging in mouse models indicated potential applications for several diseases of the nervous system, including glioma and stroke.^60^ The promising performance^54^ of this *ex vivo* tissue imaging approach for intraoperative brain tumor histology prompted the development an operating room compatible SRS microscope based on a portable fiber-laser.^61^ The authors imaged unprocessed excised specimens from 101 neurosurgical patients using stimulated Raman histology (SRH) and were able to generate virtual staining that recapitulated hematoxylin and eosin (H&E) stain appearance with essential diagnostic features. These images, generated using unprocessed surgical specimens, when compared with conventional tissue staining protocols, provided concordance for over 92% diagnostic accuracy. A further benefit of this stain-free, real-time imaging approach is the potential for automated image classification for diagnostic prediction. Capitalizing on machine learning techniques, in this case a multilayer perceptron, the classifier was trained with image attributes automatically extracted from over 120 000 fields of view (FOVs) and comprises a proof-of-principle demonstration of automated diagnostic predictions.

**Fig. 5:**
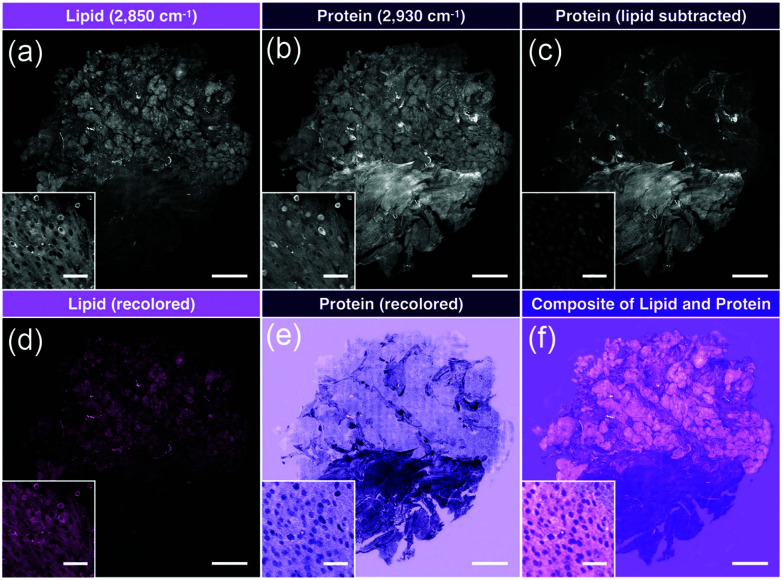
Image processing of stitched SRS imaging data (Meningioma, WHO grade I). (a) Stitched field-normalized data for lipid channel. (b) Stitched field-normalized data for protein channel. (c) Lipid data subtracted from protein, utilizing lipid and protein images in (a and b). (d) Recoloring result of lipid data. (e) Recoloring result of protein data. (f) Composite image of (d and e). Whole tissue scale bar: 1 mm. Inset scale bar: 50 μm. Reproduced figure from Shin *et al.*, Intraoperative assessment of skull base tumors using stimulated Raman scattering microscopy, *Sci. Rep.*, 2019, **9**, 20392, DOI: 10.1038/s41598-019-56932-8, with permission under Creative Commons Attribution 4.0 International License.^63^

A similar approach was implemented for pediatric brain tumors for quantitative histology.^62^ This study utilized a different set of extracted image features and different design of machine learning, but was able to capture diagnostic histological features to differentiate tumors of the posterior fossa. Extending this technique as a direct comparison with standard pathological evaluation for cancer subtyping, Shin *et al.* utilized simultaneous two-color SRH imaging for intraoperative assessment of skull base tumors.^63^ Using the lipid- and protein-tuned SRS imaging to generate pseudo-H&E images from fresh resected tumor tissues (Fig. 5), direct evaluation by neuropathologists indicated that SRH imaging could attain 87% diagnostic accuracy when compared with standard H&E from formalin-fixed parrafin-embeded (FFPE) samples. Furthermore, for cases that were challenging for SRH pseudo-H&E image rendering, the addition of chemical information obtained *via* SRS measurement could aide in diagnosis without the need to turn to alternative staining protocols.

Most recently, an automated tissue-to-diagnosis pipeline based on deep learning and two-color SRH imaging of brain tumors was evaluated in a multi-center trial.^64^ Unlike in their previous work, here the authors utilized a convolutional neural network (CNN) to train an automated image feature extractor based on a library of 2.5 million SRH images. To test the developed CNN for 13 histological classes, imaging was performed at three tertiary medical centers in a randomized clinical trial comparing pathologist interpretation of standard H&E histology with CNN-based interpretation of SRH images from small excised tissue specimens. Both the control and experimental arms of the study achieved nearly 94% diagnostic accuracy, demonstrating the potential for SRH with deep learning diagnosis to improve intraoperative decision making for neurosurgical oncology.

Similar methods using frequency modulation SRS^65^ and the combination of deep learning with SRS microscopy^66^ have been utilized for gastrointestinal and laryngeal tumors, respectively, in addition to other disease targets; however, these demonstrations were simulations of intraoperative performance and have yet to advance to the stage of intraoperative SRH. However, with continued instrumentation and analytical development, these and many other applications will be likely targets for intraoperative investigation in the near future.

### 3.2 Cutaneous coherent Raman imaging

The value of rapid, *ex vivo* CRI histology may improve the evaluation of excised specimens and enable automated diagnosis for disease states such as cancer. Given that these images can be acquired from unprocessed tissues with no additional contrast, are inherently three-dimensional due to the nonlinear optical excitation, and are non-destructive in nature, an overarching goal is to directly apply CRI to native tissues *in situ* within a clinical setting or operating theater. This translation of the complex instrumentation for clinical compatibility is further complicated by the methods needed to interact with the tissues of interest, however, several groups have explored new instruments and implementations to enable *in vivo* CRI.

**Fig. 6:**
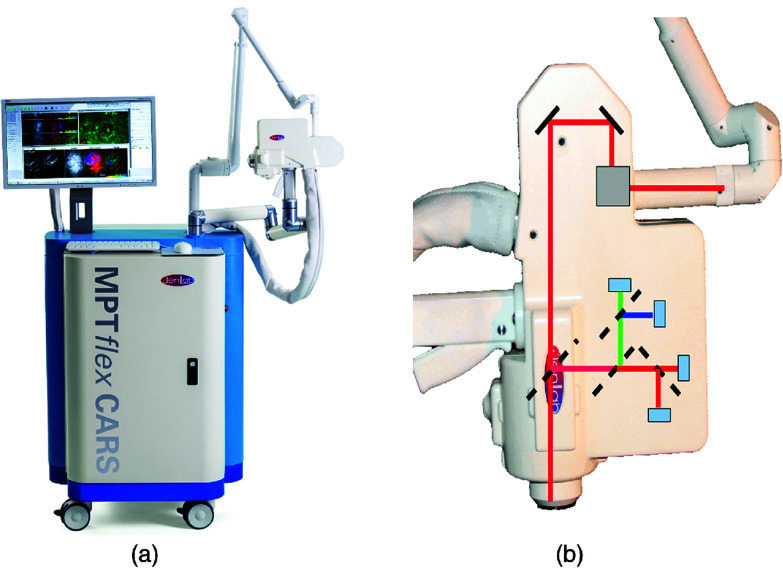
(a) Clinical multiphoton tomography MPT-CARS system (from JenLab GmbH) for concurrent acquisition of two-photon autofluorescence, FLIM, collagen imaging by SHG, and lipid/protein imaging by CARS. (b) The 360° measurement head contains four detectors. Reproduced from König *et al.*, Translation of two-photon microscopy to the clinic: multimodal multiphoton CARS tomography of *in vivo* human skin, *J. Biomed. Opt.*, 2020, **25**(1), 014515, DOI: 10.1117/1.JBO.25.1.014515, with permission under Creative Commons Attribution 4.0 International License.^73^

**Fig. 7:**
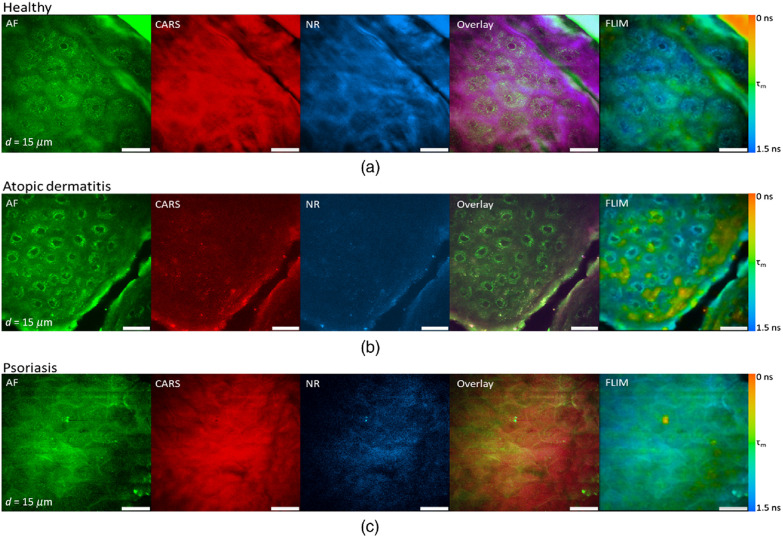
Clinical multimodal imaging of human skin *in vivo* depicted as image planes extracted from a 3D stack of an optical biopsy of the arm of (a) a healthy patient compared to the skin of patients suffering from (b) atopic dermatitis (AD) and (c) psoriasis. The reported approach enables complementary contrast acquisition; AF: autofluorescence; CARS: coherent anti-Stokes Raman scattering; NR: nonresonant background; FLIM: fluorescence lifetime imaging (*τ*_m_ = mean lifetime). Reproduced from König *et al.*, Translation of two-photon microscopy to the clinic: multimodal multiphoton CARS tomography of *in vivo* human skin, *J. Biomed. Opt.*, 2020, **25**(1), 014515, DOI: 10.1117/1.JBO.25.1.014515, with permission under Creative Commons Attribution 4.0 International License.^73^

The typical CRI microscope is based on one or more large footprint laser sources and a dedicated laser scanning microscope system. These systems are generally incompatible with the space restrictions and mobility requirements necessary for clinical translation. Furthermore, whether based upon an inverted or upright design, these microscopes offer limited options for interacting with tissues *in situ*. Despite the numerous technical and translational challenges, early work adapting multiphoton tomographic systems for CARS measurements in human skin was first reported by König *et al.* This initial system was based upon a tunable NIR femtosecond laser oscillator combined with an optical parametric oscillator (OPO) to achieve synchronized *ω*_pump_ and *ω*_Stokes_ pulses.^67^ Using low total excitation power at the focus of a high NA objective, femtoliter volumes within the skin were imaged based on beam scanning. The initial demonstration, tuned to the 2845 cm^−1^ CH_2_ stretching vibrations, simultaneously acquired two photon excited fluorescence (TPEF), second harmonic generation (SHG), and CARS images from healthy control skin, psoriatic lesion skin, and skin following the topical application of omega-3 oil, with a clinically-compatible, albeit large, instrument. Further reports from this same group have expanded the demonstrated dermatologic targets to include multiple depths (stratum corneum, stratum spinosum, stratum basale), diseases (squamous cell carcinoma, atopic dermatitis), and technical improvements to enhance flexibility (Fig. 6), decrease size, and acquire other optical modalities, such as fluorescence lifetime (FLIM) and multiplex CARS, as well (Fig. 7).^67–73^

These approaches have all utilized CARS imaging; however, the confounding non-resonant background signals that coincide with CARS imaging and the quadratic dependence of CARS signal with the concentration of the Raman scatterer are limitations for this approach. Stimulated Raman scattering, conversely, is background-free and exhibits linear concentration-dependent signals. Despite these features, there are, as yet, no *in vivo* devices for SRS imaging due, in part, to the low levels of backscattered near infrared laser light. In fact, the only report of *in vivo* SRS images acquired from human skin was obtained from the arm of a healthy human volunteer after the application of deuterated DMSO with a unique spectral peak (2120 cm^−1^).^58^ In this work, the authors utilized a large area photodiode in front of the laboratory microscope objective near the tissue interface to collect the multiple linearly-scattered photons back-propagating though the skin, coupled with custom lock-in amplifiers for the high-frequency modulated laser. The combination of technological challenges associated with *in vivo* SRS detection make the implementation of this technique non-trivial, however, the sensitivity and background-free nature of this CRI modality holds significant potential and will be the focus of on-going investigations.

### 3.3 Efforts in miniaturization and portability

Much like the tools developed to interface spontaneous Raman systems with tissues, CRI methods continue to see development in designs to interface with the patient. Improvement from large, rigid systems such as those first reported in 2011 by König *et al.* have utilized flexible, articulated arms and condensed the optical layout of the devices into more readily positioned tools that can accommodate human subjects and tissues in a variety of positions (Fig. 6). Another advancement that has helped facilitate clinical translation of CRI tools is the utilization of optical fibers for light transport, obviating the need for rigid, free-space beam paths. For many point-based translational biophotonics technologies, the primary method by which light is delivered to or detected from tissues for *in vivo* measurements is by coupling through optical fibers, improving flexibility and versatility for practical clinical applications. However, as two ultrafast laser pulses of different wavelengths utilized for CRI are also known to generate nonlinear interactions in the fiber optic core materials (often glass or crystalline), conventional optical fibers exhibit many drawbacks for remote imaging applications for CRI.^74^

Capitalizing on the developments of optical fiber technologies compatible with ultrafast laser pulses, several groups have made efforts to develop CRI tools for portability, with handheld tissue interface devices that could one day be applied to *in vivo* clinical applications. The general decrease in size for the optical components is paired with challenges for miniaturization, potential for increased system aberrations, and limitations in performance: efficient fiber delivery of ultrafast laser pulses, efficient fiber optic collection for CRI signals, miniaturized devices for laser scanning and image formation, and design of miniature optics for high resolution imaging that are aberration corrected. Many of these efforts have focused on the excitation and image formation with optical fibers, yet have still relied upon conventional optical tools such as galvanometric scanners and commercial microscope objectives. Despite these challenges, Murugkar *et al.* took an alternate approach to develop a miniaturized handheld multimodal CARS, TPEF, and SHG microscope based on a microelectromechanical system (MEMS) two-dimensional scanning device and custom designed miniaturized probe for nonlinear imaging.^75^ Utilizing computer-aided design for the beam expander and collection optics within the custom-designed barrel, the system uses fast 2D scanning from the MEMS mirror with a 0.6 NA lens to achieve a 100 × 100 μm field of view and a 400 μm working distance. Chromatic aberrations between the pump and Stokes excitation pulses were compensated for using multiple optics and two differing glass types, ultimately yielding a 1.8 mm diameter barrel that was sealed with a thin glass window for immersion imaging. The silicon MEMS mirror employed was coated with aluminum for enhanced reflective performance in the NIR, and resonant operation was controlled using electronics to sweep the fast and slow axes of the mirror. The use of the MEMS mirror introduced a conical distortion in imaging, as the authors expected from the relative angle between the slow axis of the MEMS mirror and the incident beam, however, they suggested this could be corrected with appropriate post processing. This group further developed this work, ultimately culminating in the development of a multimodal CARS exoscope with the potential for non- or minimally-invasive *in vivo* imaging.^76^ This proof of principle work utilized a table top laser source and fixed or fresh rodent tissues for demonstrative purposes of TPEF, SHG, and CARS imaging; however, the development of such a handheld multimodal nonlinear imaging tool is indicative of the ongoing efforts to improve the flexibility and decrease the size of these technologies toward versatile translational devices.

In order to build a truly portable system, however, the light source for coherent Raman imaging must be built to meet and withstand the challenges of the clinical environment. Systems must be mobile, rollable, and bump-proof, with turn-key operation across a range of temperatures and humidities. Traditional ultrafast laser systems are based on free-space optics and make use of components such as mirrors and mirror mounts that can be sensitive to motion, mechanical vibration, and temperature changes. For this reason, many of the light sources used in laboratory CRI imaging have been restricted to laminar flow floating tables within temperature-controlled laboratory spaces. Fiber laser technologies offer considerable promise for building fully portable CRI systems, as they can be made relatively insensitive to mechanical shock and temperature changes. Engineering efforts led by Freudiger *et al.* developed a two color synchronized picosecond laser system for CARS and SRS^77^ that was subsequently improved and built into the clinical histopathology system offered by Invenio Imaging.^60^ This effort opened the doors for further work in the coherent Raman community, with multiple fiber lasers demonstrated.^61,62,64,78^ While the rate of progress has been impressive, many of these systems have not yet been able to access vibrations across the Raman spectrum. Efforts beginning in the Fallnich lab, and now commercialized by Refined Laser Systems GmBH, led to the creation of a robust fiber laser for CRI capable of rapid tuning across the Raman spectrum in microseconds.^79^ This light source, which is portable and resistant to mechanical shock, can fit in a standard rack mount and holds promise for incorporation into portable CRI systems.

### 3.4 Endoscopic coherent Raman imaging

Optical microscopy tools, such as CRI, excel at high resolution imaging in relatively shallow depths. CRI requires both the penetration of light into tissue, as well as a tight focus to bias the nonlinear process. This has made applications of CRI in dermatology relatively straightforward, given both the relative ease of access to tissue as well as the depth of many skin structures of interest. To reach within the body, endoscopic approaches, in particular fiber optic endoscopic methods, are required.

Compared to free-space optical systems for non-linear optical microscopy, the nascent field of fiber optic nonlinear optical microscopy is still in a period of exploration and discovery. Many of the same challenges that have existed for over a decade in this field^80^ still hinder development and translation. There remains a need for stable ultrashort pulsed laser excitation sources and means to efficiently deliver ultrafast pulse train light to distant locations in the body without pulse and wavelength perturbations. Generating images *in situ* remains challenging in small endoscopic form factors, requiring laser scanning minimization to the millimeter scale with scanning speeds faster than the dynamics of the target systems to monitor changes characteristic of biological processes. Finally, there also must exist efficient methods to acquire the scattered light signal for detection. All of these features must be compatible with optical system designs that can maintain flexibility while still being compact enough to interface with existing medical equipment and interrogate organs and tissues within the body. Thankfully, developments in CRI share common needs with other nonlinear imaging modalities including TPEF, three photon excited fluorescence (3PEF), SHG, and third harmonic generation (THG), making work across these fields highly synergistic.

One of the primary fields of development that continues to drive progress towards CRI endoscopic imaging is advancement in optical fiber technology. As a relatively flexible and compact medium for light transport, optical fibers have long been used with biomedical imaging systems spanning a range of complexities.^18,80^ Conventional single mode fibers (SMF) have been the choice for imaging as they enable optical coupling with near diffraction limited spot sizes by allowing propagation of only a single spatial mode which improves image formation. However, this performance is paired with lower NAs and smaller core diameters which confer reduced light collection efficiency in comparison to multimode optical fibers. Early work in the development of CARS endoscopy by Légaré and Evans demonstrated the use of a step index silica SMF to couple 5–7 ps laser pulses which enabled excitation and image acquisition of 0.75 μm polystyrene beads based on the *ν*_s_ CH_2_ stretching mode (2845 cm^−1^).^74^ Here, the authors calculated the threshold power limits for self-phase modulation and self-focusing; utilizing excitation powers below this threshold, a 1 m SMF was selected for this proof-of-concept investigation as they noted that longer fiber lengths induced significant spectral broadening. The spatial resolution obtained by this system was defined by the focusing optics selected, and efficiently collected epi-CARS signals from small (0.75 and 5 μm diameter) beads. However, multiply forward scattered CARS signals, which are a primary source of contrast in thicker samples such as tissue, were not captured due to the SMF acting as a confocal pinhole. Despite the insensitivity to forward scattered CARS signals due to the collection optics used, this work fundamentally demonstrated the potential for CARS endoscopy, yet highlighted the limitations for imaging within deep tissue cavities, as only 1 m of optical fiber was used here and greater SMF lengths induced imaging aberrations.

Adapting the initial system developments reported for SRS imaging, Saar *et al.* was further able to demonstrate the design and testing of a combined CARS and SRS endoscope system for tissue imaging.^81^ Here, the authors coupled both pump and Stokes beams through a 1 m polarization maintaining fiber and the image was scanned using a miniature spiral scanning piezoelectric actuator. Light delivery into the tissue was achieved through a gradient indexed (GRIN) objective lens at the distal tip of the fiber and collected using a 10 mm × 10 mm photodiode at the tissue interface. Similar to the only report of epi-collection SRS imaging in human skin, this approach requires a large detection surface at the tissue interface to collect sufficient multiply-forward scattered SRS photons to generate images, and demonstrated depth sensitive image acquisition for lipid and protein structures in mouse skin and hair. This work further highlights the challenge for adequate detection of nonlinear coherent Raman scattering signals for endoscopic application, as the detector element utilized here is not compatible with the accessory channels of conventional endoscopes, indicating the need for further development towards clinically relevant designs for endoscopic SRS imaging.

In an effort to achieve endoscope designs with multimode fibers that typically exhibit reduced self-phase modulation and increased coupling efficiency,^82^ Wang *et al.* implemented a four wave mixing (FWM) suppression approach^83^ that enabled the delivery of pump and Stokes light as well as the collection of CARS generated signals using a single multimode fiber.^84^ In this and subsequent reports from the same group, the pump and Stokes beams were coupled into the single mode fiber at distinct polarizations, then combined at the distal tip of the fiber prior to sample illumination and paired with an array of detection fibers for improved collection efficiency.^85^ Other methods, using GRIN lenses with multicore imaging fibers^86,87^ or multimode GRIN fibers,^88^ have recently been reported as well. These approaches enabled the use of laser scanning or spatial light modulators proximal to the fiber in order to achieve the desired focus at the distal fiber tip, and were capable of image formation with reasonable SNRs. While the frame rates achieved in these reports are still slow compared to the speed of some dynamic biological processes, these initial demonstrations are promising developments towards fiber based *in vivo* CRI and multimodal endoscopy systems.

These studies applied single and multi mode fibers for CRI, however, they were limited to a fiber length of roughly 1 m to mitigate nonlinear processes that degrade image formation. In the case of nonlinear optical excitation in longer fibers, the performance of single mode fibers significantly hinders excitation and image formation. Nonlinear excitation pulses (often ≥80 fs to ≤7 ps in duration) are altered through spectral and temporal broadening from group-velocity dispersion, self-phase modulation, and potentially cross-phase modulation (XPM) if two colors of pulse trains are co-propagated. Advanced optical fibers based on structured crystal arrays (photonic crystal fibers, PCFs) have improved the transport performance of light delivery for nonlinear optical microscopy, enabling endlessly-single mode delivery of ultrashort pulses.^80^ An important consideration, especially when economical use of space is required, such as with flexible endoscope design, is that copropagating laser pulse trains are capable of generating FWM signals at the CARS frequency which can interfere with image extraction, and may necessitate the use of separate excitation and collection fibers.^89^

**Fig. 8:**
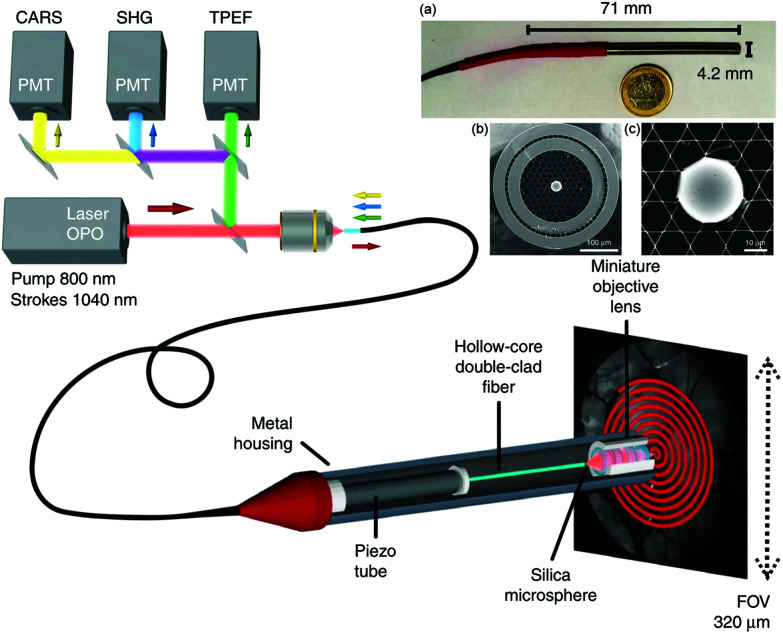
Coherent Raman endoscope design based on a dual output tunable femtosecond laser. The pulses are combined and injected into the hollow-core fiber probe to perform simultaneous CARS, SHG, and TPEF endoscopy. The light emitted by the sample is epi-collected through the same 1 m long hollow-core fiber before detection *via* photomultiplier tubes (PMTs). (a) A picture of the endoscope probe head inserted into its stainless steel housing. (b) Scanning electron microscope image of the sealed Kagomé hollow core fiber with inserted 30 μm silica microsphere, with (c) close-up view of the inserted microsphere. Adapted figure from Lombardini *et al.*, High-resolution multimodal flexible coherent Raman endoscope, *Light: Sci. Appl.*, 2018, **7**, 10, DOI 10.1038/s41377-018-0003-3, combining Fig. 1 and 3 with permission under Creative Commons Attribution 4.0 International License.^91^

Development of a double-clad hollow core PCF by Brustlein *et al.* marks one of the advancements in fiber technology with potential to mitigate some of the effects of nonlinear excitation for CRI.^90^ Here, by using pure fused silica in a stack and draw process, custom optical fibers were produced that surrounded a conventional hollow core PCF design with an outer annular silica waveguide for coupling of collected coherent Raman signals. As the excitation light is confined to the hollow core PCF and the multiply scattered light generated in the sample is mostly coupled through the annular silica waveguide, this design was capable of both CARS and SRS collection. Similar to the larger surface area for multiply scattered SRS signals previously reported by the Xie group, the inclusion of a larger source-detector offset in this fiber design aided in the collection of SRS signal, and indicates the potential for further improvement and integration for CRI fiber endoscopes. A more recent development using a double cladding hollow-core fiber with a Kagomé-lattice and an embedded microsphere was further reported by this same group (Fig. 8).^91^ Integrating a miniature piezo scanner, a small rigid endoscope fiber of 4.2 mm outer diameter and 71 mm rigid length was fabricated with the capabilities to perform CARS, SHG, and TPEF endoscopy with submicrometer spatial resolution. The hollow core fiber design suppresses FWM and XPM, with the system demonstrated with *ex vivo* tissues for multimodal nonlinear endoscopy imaging, showcasing the potential for clinical translation of such a device.

Some of the same technological developments for flexible CRI endoscope systems have been adapted for rigid endoscope designs to obtain high resolution imaging with large fields of view. Zirak *et al.* fabricated a custom GRIN lens based design that could achieve spatial resolutions on the order of 750 nm across roughly 250 μm field of view.^92^ This air-spaced, 0.5 NA imaging system design provided good performance in bulk tissues as well as relatively thin samples, and had the capability for multimodal imaging with both CARS and TPEF, but the optical components required a rigid probe tip that was 2.2 mm in diameter yet 187 mm in length, potentially limiting utility for some *in vivo* imaging applications. With an aim towards robot-assisted surgery for intraoperative nerve visualization, Hirose *et al.* used a single mode fiber to couple laser light into a rigid endoscope unit with integrated galvanometric scanning through a pair of relay lenses mounted in a 12 mm diameter, 270 mm long tube.^93^ The use of free space optical components enabled CARS imaging with a large field of view (≥650 μm diameter) with high uniformity and minimal distortion. Despite this performance, long imaging times were required, but the authors reported that performance could be improved by controlling chromatic aberrations, laser spot sizes, and focal spot sizes. The ability to optimize system designs to meet application specific needs will be crucial for these technologies to impact clinical medicine and surgery in the years to come.

## 4 Outlook and conclusions

Raman tools offer considerable benefits in both current and future clinical applications. By providing chemically-selective contrast without the need for labels *in situ*, Raman approaches have the potential to provide new diagnostic information, guide therapeutic planning and outcomes, and open up new areas of investigation. The pace of innovation, particularly in the coherent Raman imaging community, has been rapid over the past decade, with new advances in fiber lasers, fiber optics, and computational resources driving the translation of Raman spectroscopic imaging tools.^94,95^ These innovations in the hardware, the analysis, and the medical application space continue to enhance the versatility of Raman scattering based methods to achieve the high-speed imaging that is needed for clinical applications.

As optical tools, Raman methods have the advantage of being complementary to a wide range of existing optical imaging and spectroscopy methods. For example, Raman spectroscopy is routinely paired with white light and fluorescence imaging, while coherent Raman imaging can readily be carried out simultaneously with multiphoton fluorescence and harmonic imaging. This complementarity has allowed a myriad of imaging studies utilizing different contrast mechanisms that can provide prompt information to clinicians. This is of great importance when introducing new imaging tools into the clinical workflow, as physicians, nurses, and caregivers are already trained and proficient with numerous existing tools. Pairing Raman imaging methods with well-known, established imaging approaches, such as white light or fluorescence contrast, has the capability of leveraging existing expertise and familiarity to build and augment future clinical tools. In dermatology, for example, there has been a growing number of physicians trained in the use of reflectance confocal microscopy (RCM) for the diagnosis of skin lesions. It is relatively easy to build both CRI and RCM into the same device, with RCM contrast providing an important gateway and guide for dermatologists interested in using coherent Raman methods.

While this review has largely focused on optical hardware, equally important for clinical translation is the software that not only runs clinical devices but also drives the analysis of the generated image data. Spectral data is inherently high-dimensional and as such Raman scattering methods have led to the adoption and continued development of advanced chemometric methods to analyze both point-based and imaging data.^96–100^ With the continued increases in imaging speed, unprecedented amounts of data are being generated by biomedical studies visualizing and quantifying dynamic processes. There are vast opportunities in the acquisition and analysis of Raman data. For example, a time-lapse hyperspectral volumetric coherent Raman dataset, such as those acquired with devices developed in the Cheng lab,^101^ can be hundreds of gigabytes in size and require considerable computational power to extract meaningful information. For the Raman community, developing analytical tools that are suited for overwhelming amounts of data, for storage, processing, and analysis, will required retooling of many of the advanced analytical approaches that have been employed thus far. There has been considerable progress integrating artificial intelligence and machine learning methods into pipelines in recent years. These automated data analysis approaches that leverage machine learning will likely be extremely important in sifting through such high dimensionality clinical datasets.^64,102^ Efforts thus far are very much the tip of the iceberg: major advances in Raman data analysis techniques have begun to focus on handling the volumes of high-dimensional data generated during a single experiment, yet the expected developments that continue to focus on these efforts in the near-term will drive future clinical translation.

There is also a similar drive in Raman imaging to “do more with less”. Traditional Raman spectroscopy has relied on the acquisition of high resolution, low signal-to-noise spectra that can either be interpreted or quantified *via* chemometric analysis methods. In a similar vein, many of the hyperspectral CRI techniques developed also acquire continuous spectra, albeit over relatively narrow spectral ranges. These approaches, however, contain considerable extra information, as the spectra are essentially over-sampled depending upon the complexity of the samples and the correlations between distinct Raman features. Work to optimize data collection has been a topic of great interest for decades, attempting to enhance data acquisition speeds by ignoring redundant or non-informative information. Laboratory based methods to selectively acquire spectral data while still capturing salient differences within a sample have been performed using a variety of approaches including multivariate optical computing,^103^ compressive sensing,^104,105^ and low spectral resolution.^106,107^ Other approaches have sought to use hyperspectral Raman image content to efficiently collect sparse spatial features of a sample^25,101,108–111^ while still providing biochemical contrast. Further efforts combining both spatial and spectral sparse sampling have been demonstrated as well^112^ as a method to acquire Raman spectral imaging data in a rapid and efficient manner. Each of these techniques seeks to maximize the sample information obtained for either spectroscopic or imaging methods in the least amount of time, a necessity for the translation of clinical tools. These developments in instrumentation and algorithms for determination of sparse sampling patterns have also led to investigations of numerous analytical methods to determine efficient sparse spectral feature selection by which to sample Raman spectral information with the goal of reducing data acquisition times for both laboratory and clinical applications.^113–117^ Continued development of these approaches for both spontaneous and coherent Raman imaging methodologies may enable selective sparse spectral or spatial tools that are capable of acquiring efficient hyperspectral Raman data at sufficiently high speeds to visualize and quantify dynamic processes in cells and tissues and may offer the ability to translate Raman tools for applications requiring rapid time resolutions including pharmacology, histology, or surgical guidance.

Beyond these technological leaps, there certainly exist many engineering and regulatory barriers that must be overcome to truly translate Raman imaging methods to the clinic. In some ways, adoption of Raman methods for clinical utility has been limited due to applications – there has yet to be a break-through use for Raman spectroscopy or imaging into the clinical or surgical space. Advances in spontaneous or coherent Raman histopathology may prove to be this tipping point, however continued development across the broad application space may yet identify another candidate use. This push will hopefully be both facilitated by and drive the development of technology, which will in-turn help to expand developers of optical hardware and democratize techniques by making instrumentation more affordable. The opportunity to obtain continued funding from government and private agencies to develop and apply Raman imaging tools to address major needs in the healthcare sector is extremely important. Continuing to push these entities to prioritize optical and imaging technologies to solve biomedical problems must be a point of emphasis for academic, industry, and professional organizations alike. The focus of the translational biophotonics community must be to continue to creatively apply these tools to solve health challenges that impact the healthcare economy while improving patient care, outcomes, and quality of life. Especially given the sensitivity of these tools for such a wide array of biomedical applications, making Raman imaging systems affordable and translatable for global healthcare is a major hurdle that requires the attention of the entire community, but has not been widely addressed. In fact, most current Raman imaging devices are bespoke creations, which is ideal for early phase development, but makes eventual clinical translation challenging. Working groups will need to be formed to establish clear standards and benchmarks for devices and analysis methods following, for example, the path laid out by optical coherence tomography (OCT) working groups and standardization protocols that paved the way for the many current clinical uses of OCT. This step will require buy-in from academic leaders, engineers, industry partners, government agencies, and regulatory bodies. For any major use in the clinic, Raman imaging methods will need methods of payment and reimbursement. The recent success of RCM gaining CPT codes shows that such an approach is not only possible – it is absolutely necessary to bring new diagnostic imaging methods to the bedside.

## Conflicts of interest

CLE is an inventor on patents for CARS microscopy that have been licensed to multiple microscope manufacturers.

## Supplementary Material
